# Cord Blood Glutathione Depletion in Preterm Infants: Correlation with Maternal Cysteine Depletion

**DOI:** 10.1371/journal.pone.0027626

**Published:** 2011-11-16

**Authors:** Alice Küster, Illa Tea, Véronique Ferchaud-Roucher, Sabrina Le Borgne, Claire Plouzennec, Norbert Winer, Jean-Christophe Rozé, Richard J. Robins, Dominique Darmaun

**Affiliations:** 1 UMR Physiologie des Adaptations Nutritionnelles, INRA, Université de Nantes, Nantes, France; 2 Division of Neonatology, CHU de Nantes & INSERM, Centre d'Investigation Clinique, Nantes, France; 3 CNRS, CEISAM, UMR 6230, Université de Nantes, France; 4 Division of Obstetrics, CHU de Nantes, & INSERM, Centre d'Investigation Clinique, Nantes, France; 5 Mass Spectrometry Core Facility, CRNH, Nantes, France; 6 Research Laboratory, Nemours Children's Clinic, Jacksonville, Florida, United States of America; Institute for Clinical Effectiveness and Health Policy (IECS), Argentina

## Abstract

**Background:**

Depletion of blood glutathione (GSH), a key antioxidant, is known to occur in preterm infants.

**Objective:**

Our aim was to determine: 1) whether GSH depletion is present at the time of birth; and 2) whether it is associated with insufficient availability of cysteine (cys), the limiting GSH precursor, or a decreased capacity to synthesize GSH.

**Methodology:**

Sixteen mothers delivering very low birth weight infants (VLBW), and 16 mothers delivering healthy, full term neonates were enrolled. Immediately after birth, erythrocytes from umbilical vein, umbilical artery, and maternal blood were obtained to assess GSH [GSH] and cysteine [cys] concentrations, and the GSH synthesis rate was determined from the incorporation of labeled cysteine into GSH in isolated erythrocytes *ex vivo*, measured using gas chromatography mass spectrometry.

**Principal Findings:**

Compared with mothers delivering at full term, mothers delivering prematurely had markedly lower erythrocyte [GSH] and [cys] and these were significantly depressed in VLBW infants, compared with term neonates. A strong correlation was found between maternal and fetal GSH and cysteine levels. The capacity to synthesize GSH was as high in VLBW as in term infants.

**Conclusion:**

The current data demonstrate that: 1) GSH depletion is present at the time of birth in VLBW infants; 2) As VLBW neonates possess a fully active capacity to synthesize glutathione, the depletion may arise from inadequate cysteine availability, potentially due to maternal depletion. Further studies would be needed to determine whether maternal-fetal cysteine transfer is decreased in preterm infants, and, if so, whether cysteine supplementation of mothers at risk of delivering prematurely would strengthen antioxidant defense in preterm neonates.

## Introduction

Premature birth abruptly propels the fetus from the protected, relatively hypoxic intrauterine milieu into an environment with a high oxygen pressure, and further exposes the preterm infant to free radical injury from mechanical ventilation strategies, including the use of high inspired oxygen fractions or inhaled nitric oxide, and from reactive oxidative species (ROS) produced by sepsis and/or inflammatory response. Physiological perturbation is further enhanced because preterm have diminished antioxidant defenses and are highly vulnerable to oxidative stress [1]. Evidence for peroxidation of lipids and proteins is found in several tissues in premature infants [2], [3], and oxidative stress is involved in the pathophysiology of several diseases associated with prematurity, such as retinopathy, bronchopulmonary dysplasia (BPD) and necrotizing enterocolitis [4].

The tripeptide glutathione (GSH), or L-γ-glutamyl-L-cysteinyl-glycine, is the most abundant intracellular antioxidant and is actively produced in nearly all mammalian cells, where its concentration reaches the millimolar range. The maintenance of intracellular GSH stores depends on the capacity of the cell's enzymatic equipment for GSH synthesis, the availability of precursor substrates, and the cell's ability to recycle oxidized glutathione (GSSG) into its active reduced form, GSH. Depletion of blood GSH has been demonstrated in premature infants after a few days of life [5], [6], and blood GSH correlates with gestational age [5]. Cysteine availability has been considered limiting for the synthesis of GSH in other pathological situations, such as malnutrition or human immunodeficiency virus infection, since providing cysteine has been shown to stimulate GSH synthesis and restore GSH stores in such situations [7], [8].

Early in life, cysteine may be an essential amino acid, as the transsulfuration pathway producing cysteine from methionine and serine via homocysteine, is not fully active, since activity of cystathionine-γ-lyase is barely detectable in human fetal tissues [9]–[15]. Although both enterally [16] and parenterally [17] fed premature infants have been shown to be able to produce cysteine, the capacity for intrahepatic *de novo* cysteine synthesis appears to be directly proportional to the maturity of the neonate [10]. Nevertheless, providing cysteine even in high doses to preterm infants after birth seems neither to increase [GSH] nor to enhance GSH synthesis rate [18].

To obtain further insight into the mechanisms involved in the GSH deficiency observed in premature infants, we have compared the [GSH] and [cys] in umbilical cord blood from very low birth weight infants (VLBW) (gestational age (GA)<32 weeks and/or birth weight <1500g) with those of healthy full term newborns. Fractional synthesis rates of GSH, (FSR_GSH_) were also determined *in vitro* by following the incorporation of L-[^2^H_2_]cysteine into erythrocyte glutathione. All measurements were performed in blood erythrocytes obtained immediately after birth from umbilical arteries and veins and in maternal venous blood. Erythrocytes were chosen for measurements instead of plasma because of their highly active GSH synthesis contributing in large amounts besides liver to the GSH pool of the preterm. We hypothesized that the GSH depletion observed in preterm infants would already be present at the time of birth, and may be due to insufficient cysteine availability from maternofetal cysteine transfer rather than to an insufficient capacity for GSH synthesis.

## Methods

### Design

The study was registered (ClinicalTrials.gov Identifier: NCT00607061), and performed in the Department of Obstetrics of the Hôpital Mère-et-Enfant at the University of Nantes after approval by the local medical ethical committee (Comité de Protection des Personnes dans la recherche biomédicale (CPP) des Pays de la Loire). The study was investigator-initiated and funded by the Hospital of Nantes. Written, informed consent was obtained from every mother, a few hours before delivery.

Umbilical cord blood samples were collected in the delivery room simultaneously with sampling for blood gas analysis by the midwife. As umbilical cord is routinely discarded after sampling, the samples obtained for the current study did not involve any additional blood loss for the neonates.

Umbilical cord blood was obtained from VLBW infants (n = 16), and in a control group of healthy, full-term neonates (n = 16) in order to determine [GSH]. Furthermore, FSR_GSH_ were determined *ex vivo* in erythrocytes obtained from half of the babies in the VLBW group (n = 8) and compared with half of the control group of neonates born at term (n = 8). Assessment of FSR_GSH_ in umbilical cord blood erythrocytes necessitated immediate handling of samples after birth with a narrow cooperation of the neonatal care team and the GCMS laboratory team. To obtain insight into the role of the materno-fetal unit (including placenta) in cysteine and GSH exchange, all analyses were performed in blood from three sites: the umbilical vein providing information about blood directed from the placenta to the fetus; in samples collected from one of the two umbilical arteries representing blood of the fetus redistributed to placenta; and in order to relate these results to the status of the mothers, we also collected maternal venous blood samples at delivery.

### Subjects

The subjects were 16 inborn VLBW infants born before 32 weeks of GA and/or with a birth weight <1500 g, and a control group of 16 inborn neonates born at term after an uneventful pregnancy and delivery. Umbilical arterial and venous cord blood was obtained immediately after birth. Maternal venous blood was collected at delivery for every neonate included in order to obtain maternal infant pairs for comparison between the preterm delivery and the control group. In 8 VLBW infants chosen at random from the 16 subjects, [GSH] and [cys] were determined and compared with 8 healthy full term neonates also chosen at random. In the remaining groups of 8 VLBW infants and 8 full-term infants, the fractional synthesis rate of GSH, (FSR_GSH_), and [GSH] were determined in order to compare them also to 8 full-term neonates chosen at random.

Inclusion criterion for VLBW infants was a GA<32 weeks and/or a birth weight <1500 g. Inclusion criteria for the control group were: a GA >37 weeks, and uneventful pregnancy and delivery.

Exclusion criteria for the VLBW group were: perinatal asphyxia defined as pH<7.2 in umbilical cord blood and/or difference between arterial and venous pH<0.2, or major fetal pathology (abnormal karyotype, malformation, foetal pathology revealed during pregnancy).

Exclusion criteria for control group were: perinatal asphyxia, major fetal pathology, bacterial or viral infection, or maternal arterial blood pressure >90 mm Hg during pregnancy.

### Measurement of glutathione and cysteine concentrations

Using antioxidant dithiothreitol (DTT) that reduces all thiol dimers, [GSH] was measured as total GSH (reduced GSH plus oxidized GSH, the disulfide GSSG) and [cys] as total cysteine (reduced cysteine plus oxidized cystine). Hence, values include various GSH and cysteine-containing and protein-linked disulphides in addition to free monomers. Erythrocytes provided from maternal, venous, and arterial umbilical cord blood were chosen for measurements, as they are more readily available than tissue samples and have a highly active GSH synthesis with an active export towards plasma, contributing besides the liver to the extracellular GSH pool. Umbilical cord blood is moreover easy to collect and non invasive for the neonate. Determinations were performed by gas chromatography-mass spectrometry using homoglutathione as an internal standard, as previously described [19].

### Measurement of in vitro fractional rates of glutathione synthesis (FSR_GSH_)

Previous work established optimal conditions to synthesize GSH *in vitro* and thereby to determine FSR_GSH_ [19]-[21].

Immediately after sampling in delivery room, freshly sampled blood erythrocytes were washed twice by centrifugation of the blood samples and replacement of the supernatant volume-by-volume by 0.9% NaCl in order to remove enzymes contained on the surface and potentially influencing glutathione metabolism. Erythrocytes contained in the remnant phase after the last centrifugation were disrupted by adding a volume of ice cold distilled water equal to the volume of plasma discarded. A 2-mL aliquot of lysed erythrocytes was then incubated at 37°C with 2 mL of a solution (adjusted to pH 7.5 with NaOH (10 mol/L)) containing: L-[^2^H_2_]cysteine (98% enriched, Cambridge Isotopes, Andover, Mass) at a concentration of 500 µmol/L, glutamate (600 mmol/L), glycine (400 mmol/L), ATP (400 mmol/L), dithiothreitol (400 mmol/L), glucose (400 mmoL/L), MgCl_2_ (3 moL/L), and Trizma® hydrochloride (400 mmol/L). Incubation of red blood cell lysates with these adequate amounts of substrates allowed determination of maximal FSR_GSH_. 400-µl aliquots were removed at 0, 10, 20, 30, 60 and 90 min after the start of incubation. The reaction was stopped immediately by the addition of 375 µL of 50% (w/v) sulfosalicylic acid and 800 µL of 0.2 mol/L sodium phosphate buffer (pH 7.5). After centrifugation (3000×*g* for 15 min at 4°C), the supernatant was removed, filtered and each sample derivatized and analyzed by GCMS.

### Calculations

Glutathione fractional synthesis rate (FSR_GSH_, %/d) was determined from the incorporation of ^2^H_2_-cysteine into red blood cell glutathione: FSR_GSH_ = 100×24×(ΔE_GSH_/Δt)/Erbc_cys_, where Erbc_cys_ is the ^2^H_2_ enrichment in erythrocyte-free cysteine at plateau, ΔE_GSH_/Δt is the slope (MPE/h) of the regression line describing the rise in red blood cell ^2^H_2_-glutathione enrichment (MPE) as a function of time (h) over the last 3 hours of incubation, and 24 and 100 convert FSR to percent per day. FSR_GSH_ represents the fraction of glutathione pool synthesized per unit of time.

Absolute synthesis rate (ASR_GSH_, µmol/L per day) was calculated as:

ASR_GSH_  =  FSR_GSH_/100 × [GSH]

In earlier reports [19]–[21] and preliminary experiments (data not shown) we confirmed the determination of FSR to be reproducible, when it was measured in replicate samples from healthy adult volunteers, with a coefficient of variation of <5% between replicate measurements.

### Statistics

Statistical analysis was performed using SPSS® version 14.0 (SPSS, Chicago, IL). Results are reported as median and interquartiles, and percentages were presented as percent [95% confidence interval]. Mann-Whitney U and Chi^2^ tests were used to compare quantitative and proportion values, respectively. Spearman's correlation test and regression analyses were used to evaluate the relationship between maternal and venous umbilical cord blood [cys] or [GSH]. Statistically significant differences were defined as when p<0.05.

## Results

A total of 16 VLBW infants and 16 full term neonates were enrolled (**Table 1**). Half of the subjects (n = 8) in each group were randomly chosen and included for determination of [GSH] and [cys] in arterial and venous umbilical cord blood. In the second half of each group (n = 8) both FSR_GSH_ and [GSH] were determined. Both subgroups of VLBW infants were similar regarding gestational age and birthweight. All analyses were performed simultaneously in the infants' mothers.

**Table 1:** Selected clinical characteristics of enrolled infants and their mothers.

	Preterm infants	Full-term infants	
	(n = 16)	(n = 16)	p
Sex			
Male, *n*	8 (50%)	7 (40%)	
Female, *n*	8 (50%)	9 (60%)	
Birth weight, *g*	1160 [982–1317]	3490 [3305–3669]	<0.001
Gestational age, *wk*	29 [27]–[30]	40 [39]–[41]	<0.001
Birth weight, *z score*	-0.4 [−1.1–0.2]	-0.1 [−0.5–0.2]	0.29
Umbilical cord blood pH	7.3 [7.2–7.3]	7.3 [7.2–7.4]	0.14
Mode of delivery			<0.001*†
Vaginal, *n*	6 (40%)	16 (100%)	
Cesarean section, *n*	10 (60%)	0 (0%)	
Antenatal steroids	15 (94%)	0 (0%)	<0.001
Preeclampsia, *n*	6 (40%)	0 (0%)	0.02*‡
Chorioamniotitis, *n*	8 (50%)	0 (0%)	
Premature rupture of membranes (>12 hours antepartum), *n*	6 (40%)	0 (0%)	
Apgar score at 5 min	10 [8]–[10]	10 [10–10]	0.12
Infant haemoglobin *g/dL*	14.5 [13.6–16.3]	16.3 [15.0–17.0]	0.05
Maternal age, *y*	27 [24]–[34]	30 [27]–[35]	0.34
Maternal haemoglobin *g/dL*	11.6 [11.0–12.2]	11.6 [11.0–12.2]	0.17

Data are reported as n (%) or median [interquartile]. † intra-group comparison of vaginal and cesarian delivery modes. ‡ intra-group comparison of preeclampsia (Mann-Whitney U or Chi^2^ tests).

### Maternal and fetal erythrocyte GSH concentrations

**Figure 1:**
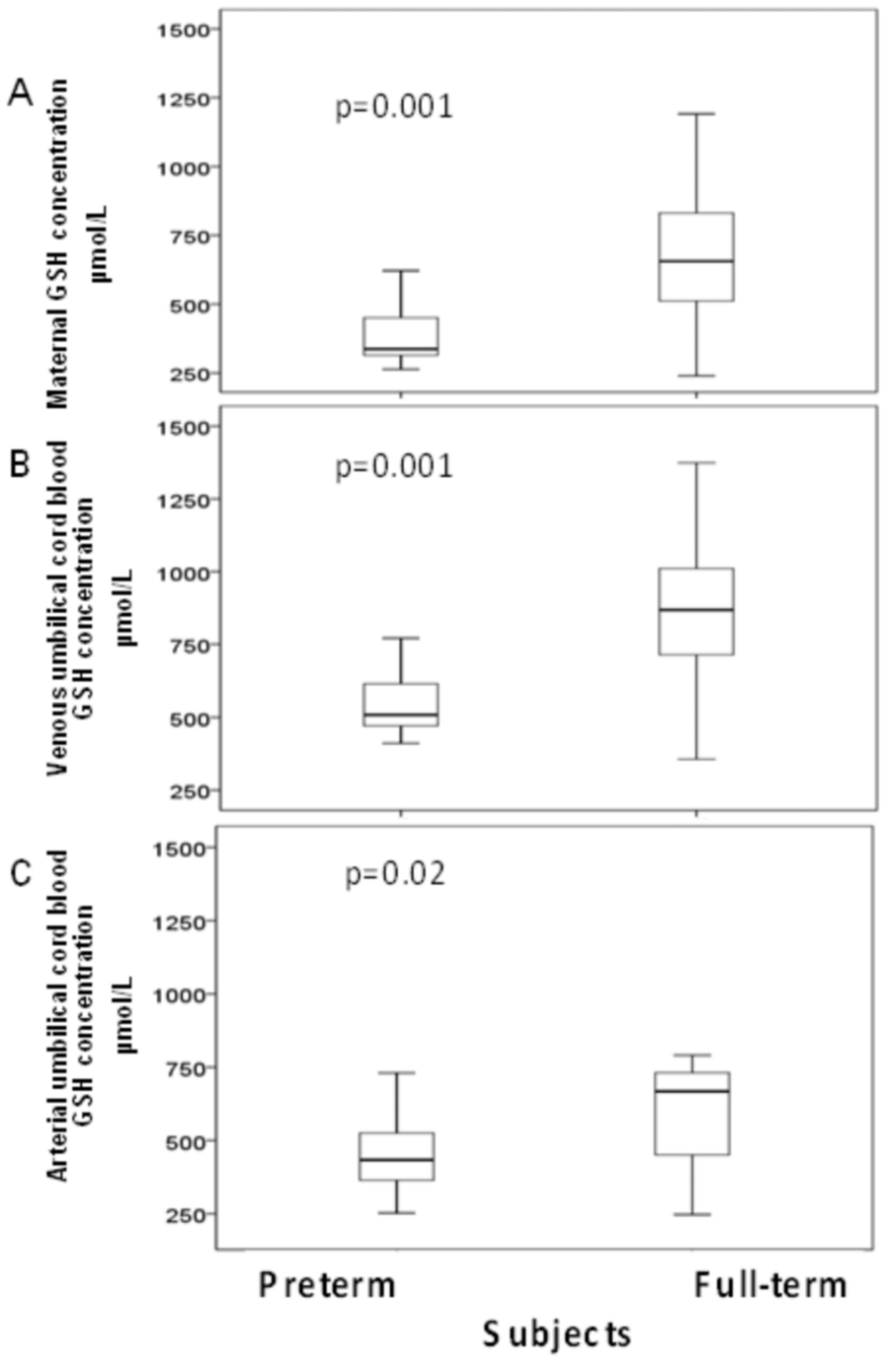
Glutathione concentration [GSH] in erythrocytes from maternal blood (A), venous umbilical cord blood (B) and arterial umbilical cord blood (C) of preterm and full-term subjects. The boxplot shows the median (central horizontal line) and includes the 25^th^ (lower box border) to 75^ th^ percentile (upper box border) of [GSH] (µmol/L). Preterm (n = 16) were compared with full-term (n = 16) subjects. Significant differences were observed between the two groups (p<0.05), as assessed with the Mann-Whitney U test.

Maternal blood [GSH] was significantly lower in mothers giving birth before term than in those who delivered at full term (median: 337 µmol/L, [interquartile, 310–479 µmol/L] *vs*. median: 656 µmol/L, [interquartile, 494–858 µmol/L]; p = 0.0003) (**Figure 1A**). Neither growth retardation nor preeclampsia (data not shown) was associated with lower maternal GSH levels than prematurity itself.

Similarly, [GSH] in umbilical venous cord blood was significantly lower in VLBW compared with full term infants (median: 509 µmol/L, [interquartile, 461–625 µmol/L] *vs*. median: 868 µmol/L, [interquartile, 711–1013 µmol/L]; p = 0.001) (**Figure 1B**). This was also the case for umbilical arterial blood [GSH] (434 µmol/L, [interquartile, 363–540 µmol/L] *vs*. 668 µmol/L, [interquartile, 450–733 µmol/L]; VLBW *vs.* full term; p = 0.02), (**Figure 1C**). Arterial umbilical cord blood [GSH] were lower than venous umbilical cord blood [GSH] and this arterio-venous gradient was larger in full-term than in premature infants (p = 0.02).

**Figure 2:**
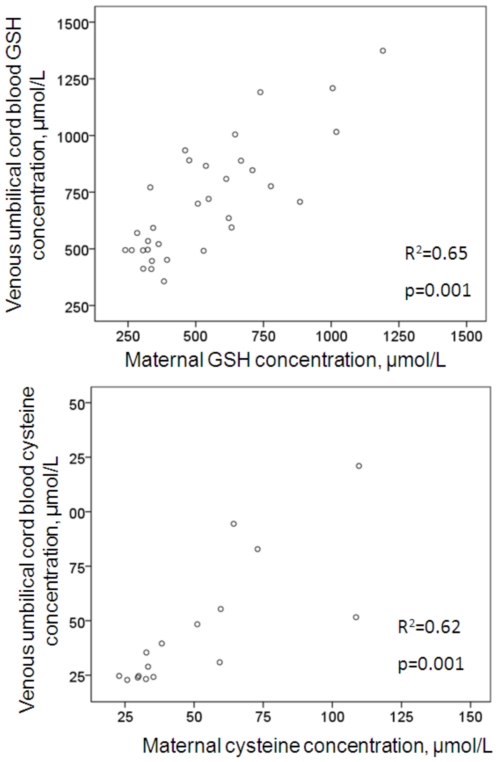
Correlation between glutathione concentration [GSH] (A) or cysteine concentration [cys] (B) in venous umbilical cord of preterm and full-term subjects and their maternal blood. [GSH] or [cys] in venous umbilical cord blood are positively and significantly correlated with those in maternal blood (R^2^ = 0.65; p<0.05 for GSH and R^2^ = 0.62; p<0.05 for cysteine) as assessed with the Mann-Whitney U test.

A strong correlation was observed between maternal and venous umbilical cord blood [GSH] (r^2^ = 0.65; p = 0.001) (**Figure 2A**).

### Maternal and fetal erythrocyte cysteine concentrations

**Figure 3:**
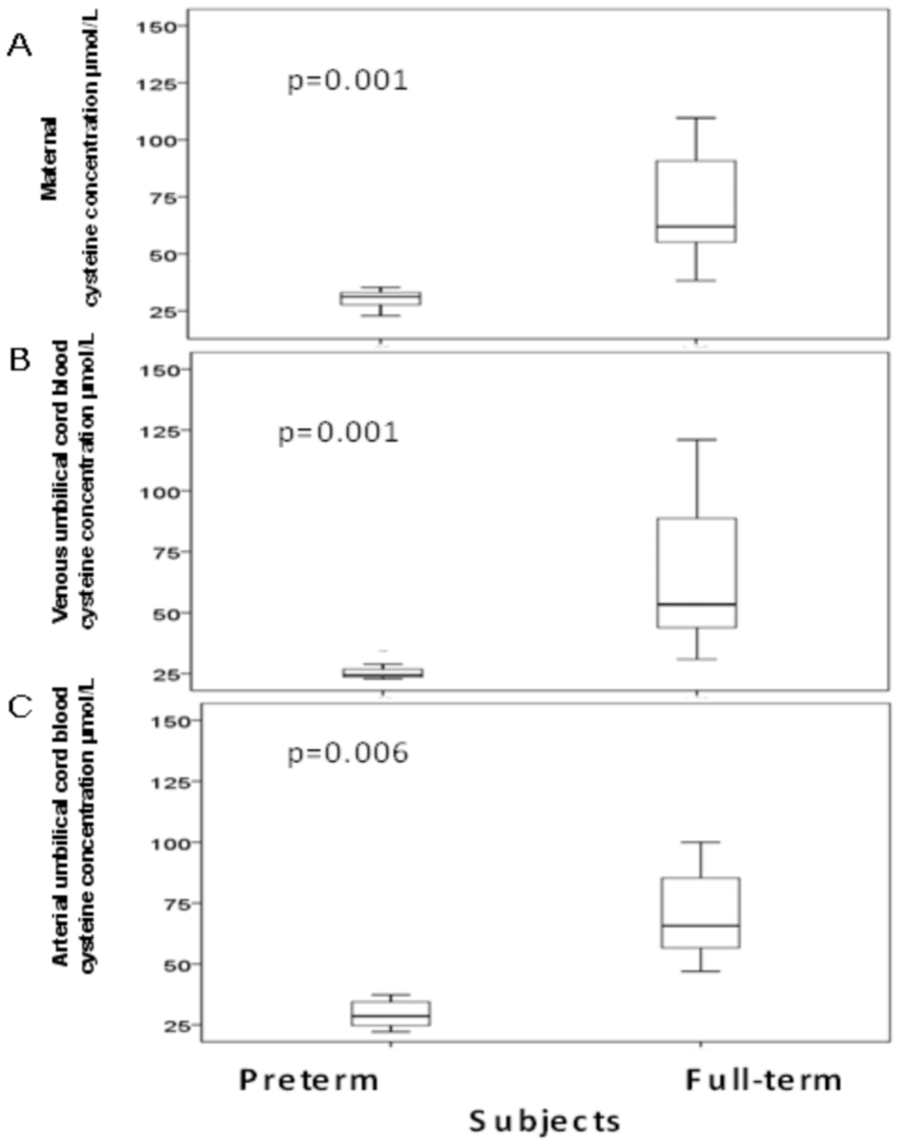
Cysteine concentration [cys] in erythrocytes from maternal blood (A), venous umbilical cord blood (B) and arterial umbilical cord blood (C) of preterm and full-term subjects. The boxplot shows the median (central horizontal line) and includes the 25^th^ (lower box border) to 75^th^ percentile (upper box border) of cysteine concentration (µmol/L). Preterm (n = 8) were compared with full-term (n = 8) subjects. Significant differences were observed between the groups (p<0.05) as assessed with the Mann-Whitney U test.

Maternal blood [cys] was significantly lower in mothers who delivered preterm, compared with full term (median: 31.3 µmol/L, [interquartile, 26.8–33.2 µmol/L] *vs*. median: 62.0 µmol/L, [interquartile, 53.2–99.7 µmol/L]; p = 0.001), (**Figure 3A**). Similarly, venous umbilical [cys] was significantly decreased in VLBW compared with full-term neonates (median: 24.4 µmol/L, [interquartile, 23.4–27.8 µmol/L] *vs*. median: 53.5 µmol/L, [interquartile, 41.7–91.6 µmol/L]; p = 0.001), (**Figure 3B**). Accordingly, a strong correlation was observed between maternal and venous umbilical cord blood [cys] (r^2^ = 0.62; P = 0.001), (**Figure 2B**).

Cysteine depletion was also found in umbilical arterial blood in VLBW infants (median: 28.6 µmol/L, [interquartile, 23.7–35.9 µmol/L] *vs*. median: 65.6 µmol/L, [interquartile, 54.0–91.6 µmol/L]; p = 0.006), (**Figure 3C**).

### Glutathione fractional synthesis rates

As shown in **Table 2**, the fractional synthesis rates of GSH (FSR_GSH_) determined *in vitro* in erythrocytes from VLBW infants did not differ from those measured in full term infants, regardless of the sampling site. When determined in arterial umbilical cord blood, there was even a (non significant) trend toward higher FSR_GSH_ in VLBW infants than in full term infants. Absolute synthesis rates (ASR_GSH_) did not differ. Similarly, the term of delivery did not affect FSR_GSH_ in maternal blood (Table 2).

**Table 2:** Glutathione FSR (FSR_GSH_) and ASR (ASR_GSH_) in umbilical cord venous and arterial blood erythrocytes of preterm and full-term infants and in erythrocytes from their respective mothers.

	Group			
	Preterm birth		Full-term birth	
	(n = 8)		(n = 8)	p
**FSR, ** ***%/d***				
Umbilical vein	145 [79–174]		98 [75–126]	0.11
Umbilical artery	234 [84–256]		93 [72–115]	0.08
Maternal vein	110 [81-238]		77 [65-115]	0.16
**ASR, ** ***µmol/L per day***				
Vein	740 [515–906]		663 [560–969]	0.79
Artery	651 [391–1222]		551 [442–634]	0.46

Data are reported as median [interquartile]. No differences were observed between the samples collected at different gestational ages (p>0.05) as assessed with the Mann-Whitney U test.

## Discussion

The findings of the current study demonstrate that blood glutathione depletion is already present at the time of birth in premature infants, compared with term infants. They further suggest that GSH depletion is not due to a defect in fetal GSH synthesis, as GSH synthetic rates measured in isolated erythrocytes were indistinguishable in preterm and term infants. Critically, evidence for a dramatic reduction in cysteine delivery to the preterm fetus is provided, with a 40% reduction in umbilical cord vein [cys] in preterm infants compared with term infants, and a 43% reduction in maternal blood from mothers delivering preterm babies. Taken collectively, the data suggest that the depletion in GSH associated with preterm birth may result from the combination of 1) increased fetal GSH utilization and 2) insufficient maternofetal cysteine delivery.

The first interesting finding in the current study was a significant reduction in maternal [GSH] in the case of preterm delivery. The effect of pregnancy on GSH status is debated, and published data concerning maternal thiol concentrations are scarce. Even though pregnancy generates increased oxidative burden, in normal pregnancy antioxidant capacity increases with term [22]. The plasma concentrations of cysteine, GSH and homocysteine are lower in healthy, pregnant women at delivery than in nonpregnant women, and plasma cysteine are lower in the 3^rd^ than 2^nd^ trimester during uncomplicated pregnancy [23]. In preeclampsia, maternal cysteine and homocysteine levels were comparable to those in nonpregnant women, but GSH levels were lower, suggesting either accelerated GSH utilization or insufficient GSH synthesis [24], [25]. In our population, preterm delivery occurred in a context of chorioamniotitis, premature rupture of membranes, or preeclampsia. All are associated with inflammation, which is known to affect redox balance [26]. In case of infection, bacterial products stimulate the release of inflammatory cytokines by decidua and fetal membranes, leading to preterm labor and generation of free radicals and ROS in fetal and maternal circulation [27]. Accordingly, premature rupture of membranes involves damage of collagen in the chorioamnion by tissue-damaging ROS molecules [27]. Finally, elevated levels of oxidized thiols have been reported in pregnancy complicated by preeclampsia [28]. A lower antioxidant capacity has been implicated in syncytiotrophoblast apoptosis and increased shedding into the maternal circulation with endothelial dysfunction that is considered a causative factor in the maternal hyperimmune response characterizing preeclampsia [29], [30]. In the current study, preeclampsia was not associated with a further decline in maternal [GSH], compared with other causes of preterm birth, suggesting depletion of GSH is present regardless of the cause of preterm delivery and due to increased oxidative stress. The majority of mothers delivering preterm underwent a Caesarean section, versus none among the mothers delivering at full term (Table 1). Although preoperative fasting or stress due to the surgical procedure might impact oxidative stress, we could not find an influence of C-section on GSH (or cysteine) levels in this subgroup. Among the mothers who delivered preterm, all but one received antenatal steroids (Table 1), and antenatal steroids were found to attenuate oxidative stress (assessed by GSH/GSSG ratio) and oxidative damage to proteins and DNA [31]. The GCMS assay employed in our study implies the reduction of GSSG to GSH and therefore cannot distinguish between the reduced and the oxidized forms of GSH and measures only total GSH. For this reason, we cannot evaluate the capacity of the mother to regenerate GSH from GSSG. Yet the lower total glutathione, combined with unaltered GSH FSR (Table 2), suggests increased rates of GSH utilization in our population of mothers delivering preterm. Finally, as we only measured glutathione FSR in erythrocytes, we cannot draw any conclusion on the synthetic rate of GSH in maternal liver. Liver indeed is the main source of plasma GSH, and hepatic GSH synthesis might be assessed through a determination of GSH synthetic rate in plasma; the assay we used is, however, not sensitive enough to measure GSH synthesis in plasma, which has a much lower GSH content (in the micro-, *vs.* the milli-molar range in erythrocytes). Erythrocyte GSH nevertheless accounts for the bulk of whole blood GSH, and may play a role in antioxidant defence even in extracellular medium [32].

In the VLBW infant group, we observed a significantly lower [GSH] determined in venous umbilical cord blood, which likely represents the sum of the GSH distributed from the placenta into fetal circulation and GSH produced by fetal erythrocytes. Even though earlier studies by Frosali et al [33] and Jean-Baptiste et al [34], found similar umbilical cord blood [GSH] between preterm and term infants, the population enrolled in the current study may not be strictly comparable, as the average birth weight was 1160g in our study, compared with 1734 and 1827 g in the studies by Frosali et al and Jean-Baptiste et al, respectively. We speculate that the slightly lower gestational age of the preterm infants enrolled in the current study may account for the difference with these earlier studies [33], [34]. In studies performed several days after birth GSH depletion was observed in preterm infants before, with the lowest GSH content observed in the less mature infants: lower [GSH] in premature than in term newborns has been found in peripheral venous blood following birth with a more pronounced decrease of lymphocyte [GSH] on day 2 from birth on [5], and in erythrocytes of preterm with respiratory distress syndrome [6]. In cells from tracheal aspirates derived from oxygen-dependant newborn infants, intracellular total [GSH] has been found lower in male and in less mature infants [35]. Another study confirmed that GSH content in tracheal aspirates from preterm with IRDS is decreased [36]. Our study is first to demonstrate that GSH depletion is present in preterm infants as early as at the time of birth as we measured [GSH] in umbilical cord blood in the delivery room. We speculate that in the very preterm infants enrolled in the current study, either (a) GSH was depleted prior to birth due to the underlying cause of prematurity, or (b) the process of birth *per se* contributed to a precipitous drop in GSH during birth, resulting in low cord blood GSH at the time of sampling.

Antioxidant reserve may be of crucial relevance at the moment of transition from fetal to neonatal life when the newborn is exposed to an oxidative environment, and GSH depletion may affect outcome, since oxidative-stress participates in the pathophysiology of necrotizing enterocolitis, retinopathy, periventricular leukomalacia and chronic lung disease [5], [33], [34]. Low plasma GSH levels during the first hours of life have indeed been found in infants with respiratory distress syndrome (RDS) [37] and in infants who later developed BPD [38].

As for cysteine, we observed cysteine depletion in VLBW infants and their mothers. The low cysteine levels observed in mothers of preterm infants are intriguing. From a theoretical standpoint, several factors could contribute to low maternal cysteine concentration, including: (a) insufficient dietary methionine intake; (b) poor conversion of methionine to cysteine; or (c) excess utilization of cysteine via cysteine utilization for glutathione synthesis or incorporation into body protein. The mothers of preterm infants had been hospitalized for an average of 10.2 days (versus 0.2 days for the group delivering at term) prior to delivery. So they ingested hospital food rather than home cooked food. The diet plan offered to the mothers was a regular hospital diet providing 2600 kcal/d with 16% protein, and an estimated methionine + cysteine content of 4200 mg/d, which exceeds the estimated requirements for adults (≈13 mg/kg/d, i.e., 780 mg/d for an adult of average body weght) [39]. However, dietary intake was not monitored through their hospital stay, and the mothers may not have consumed their entire meals, since insufficient intake has long been documented among patients during hospital stay: for instance, in a recent European multicenter study, more than half of the patients did not eat their full meal provided by the hospital [40]. Whether the conversion of methionine to cysteine operated normally cannot be ascertained, as we did not measure methionine concentration; impaired conversion is, however, unlikely as the conversion occurs in liver and liver function was not altered in delivering mothers Increased utilization of cysteine for glutathione synthesis is, however, likely as most mothers who delivered prematurely did so because of conditions known to be associated with inflammation, and inflammation invariably is associated with increased oxidative stress, which, in turn enhances glutathione synthesis. Maternal cysteine depletion therefore likely results from the underlying disease that led to preterm delivery.

Cysteine levels must be sufficiently high to meet the requirements both for protein synthesis and the production of GSH, but, conversely, must be kept below the threshold of cytotoxicity. GSH synthesis decreases with sulfur amino acid intakes that are marginal but adequate for protein synthesis, suggesting protein synthesis has a higher priority for cysteine than does GSH synthesis [41], [42].

Our results suggest that umbilical venous cord blood [cys] may directly depend on maternal levels. Energy-dependent transfer of amino acids is the preferred route for essential amino acids, with little or no metabolism within the placenta [43], whereas non-essential amino acids may be processed, interconverted or consumed within the placenta. Cysteine entry to the fetal circulation is mediated by system x_c_^-^, a transport protein which transports cysteine in exchange for glutamate. Therefore, as cysteine is the rate-limiting precursor for GSH synthesis, the intracellular level of GSH is regulated by system x_c_^-^ activity [43]–[45].

Cysteine may be a conditionally essential amino acid for preterm infants since cystathionase, a key enzyme catalyzing the last step of the transsulfuration pathway, cleaving cysteine from cystathionine, is undetectable in fetal liver, with activity appearing only postnatally [9]. In premature neonates, the low activity is associated with higher cystathionine and lower [cys] in plasma [15]. Both plasma cysteine [15] and hepatic *de novo* cysteine synthesis [10] rise in proportion to gestational age. Even though low plasma [cys] have been found in preterm infants receiving cysteine-containing parenteral amino acid solutions [46], [47] cysteine deficiency seems to be relative, since both enterally-[16] and parenterally-fed [10], [17] premature infants are able to produce cysteine. Demand may, however, exceed synthesis in case of RDS, as lower erythrocyte [cys] were observed in preterm infants on day 0, and in those who developped BPD or who died, both erythrocyte [cys] and [GSH] were found impaired on day 1 [39].

In the current study both GSH and cysteine were depleted in preterm infants at the time of birth, compared with term infants. The two substrates indeed are tightly linked. First, depletion of GSH decreases the synthesis of S-adenosylmethionine (SAM) by inhibition of L-methionine-S-adenosyltransferase [48], [49]. As SAM is an allosteric activator of cystathionine β synthase, transsulfuration is impaired and homocysteine is preferentially diverted to the transmethylation pathway [50], resulting in a lower cysteine availability for GSH synthesis. Accordingly, in a model of preterm baboon, supplementation with GSH, but not cysteine, prevented the decline in cysteine occurring postnatally [51]. Secondly, GSH is considered a cysteine reservoir, and low cysteine availability may increase GSH breakdown.

Besides cysteine, glutamate and glycine, the other two precursors of GSH, may affect fetal GSH availability. Glycine indeed is considered a conditionally essential amino acid in the neonate [52], and arises from the placental conversion of serine by serine hydroxymethyltransferase, which has a low activity in human placenta [53], [54], whereas glutamate, is known to arise from the conversion of glutamine in fetal liver [55]. As the assay used in this study was developed to specifically assess GSH and cysteine, we cannot ascertain whether glutamate and/or glycine deficiency contributed to GSH depletion in VLBW cord blood.

The FSR measured *ex vivo* in the current study are approximately 5 times higher than those measured *in vivo*, *e.g.*, 234% per day in umbilical arterial blood in preterm infants in this study, compared with approximately 45% per day in studies by Te Braake et al [56]. Several factors obviously contribute to the difference between rates measured *in vivo* and *ex vivo*: (a) the freshly collected erythrocytes are lyzed, so equilibration of substrates between extra- and intracellular milieus become immaterial; and (b) erythrocytes are incubated with very high concentrations of all glutamate, cysteine and glycine, that largely exceed concentrations occurring *in vivo*. The *in vitro* method therefore measures the maximal capacity for GSH synthesis, when the erythrocyte enzymatic machinery is experiencing unlimited supply of precursor amino acids.

In the current study, the FSR_GSH_, measured *ex vivo* in neonatal erythrocytes, was indistinguishable between preterm and term infants, and close to maternal values (Table 2). This suggests that the observed GSH depletion may be due to increased utilization and/or insufficient substrate availability rather than to immaturity of synthetic capacity. This is in agreement with the early maturation of the enzymatic activity for GSH synthesis documented in earlier studies [9], [57]. GSH is synthesized from glutamate, cysteine, and glycine in two consecutive steps by the action of γ-glutamylcysteine synthetase (GCS), which is rate-limiting, and GSH synthetase. GCS activity levels have been found in the same range in human foetal erythrocytes [58], leukocytes [57], liver [59], lung and kidney as are found in adults [60]. Short term regulation of GSH production by GCS occurs mainly via the availability of cysteine. The K_m_ of rat liver GCS for cysteine is 100 µmol/L, near the upper end of typical cellular [cys], and the rate of GSH synthesis is extremely sensitive to changes in cellular cysteine level. GSH synthesis increases when intracellular cysteine levels increase as a result of increased saturation of GCS with cysteine, and this contributes to removal of excess cysteine. When cysteine levels drop, GCS activity increases and the increased capacity for GSH synthesis facilitates conservation of cysteine in the form of GSH, although the absolute rate of GSH synthesis still decreases because of the lack of substrate. Our results showing fully efficient FSR_GSH_ in prematures are in agreement with this mechanism.

The unaltered FSR_GSH_ observed for the VLBW infants in the current study concurs with a recent study that found no difference in FSR_GSH_ on day 2 in prematures (<1500 g) receiving or not from birth an amino acid supplement in their parenteral nutrition [56]. Early amino acid infusion indeed increased blood [GSH] in VLBW infants, without any change in GSH FSR, suggesting amino acids may decrease GSH utilization, rather than increase GSH synthesis [56], [61]. Taken together, the findings from the latter study and those from the current study point in the same direction: that substrate availability may be the limiting factor of maintaining adequate [GSH] after birth. Further studies using maternal infusion of labeled cysteine may thus be warranted to quantitate the rates of maternal-fetal cysteine transfer, and establish whether such transfer is lower in VLBW than term infants. If so, the supply of additional cysteine would become seem a reasonable option. However, intervention studies with cysteine supplied postnatally, even in high doses, failed to demonstrate an impact on FSR_GSH_, nitrogen balance, or weight gain. This may be, to some extent at least, because the included infants were not critically ill, or because [GSH] was already adequate [18], [62]. As we show that deficiency is already present at the time of birth, and as [cys] drops dramatically after birth in premature infants due to either decreased cysteine synthesis, increased utilization, or excretion, the inefficacy of post-natal cysteine delivery may be due to a ‘late’ timing and it would be worthwhile to explore the putative benefit of cysteine supply prenatally, as we find [cys] to be consistently depleted in mothers of preterm infants.
